# Antiplatelet Aggregation and Antithrombosis Efficiency of Peptides in the Snake Venom of *Deinagkistrodon acutus*: Isolation, Identification, and Evaluation

**DOI:** 10.1155/2015/412841

**Published:** 2015-09-21

**Authors:** Bin Ding, Zhenghong Xu, Chaodong Qian, Fusheng Jiang, Xinghong Ding, Yeping Ruan, Zhishan Ding, Yongsheng Fan

**Affiliations:** Zhejiang Chinese Medical University, Hangzhou, Zhejiang 310053, China

## Abstract

Two peptides of Pt-A (Glu-Asn-Trp 429 Da) and Pt-B (Glu-Gln-Trp 443 Da) were isolated from venom liquor of *Deinagkistrodon acutus*. Their antiplatelet aggregation effects were evaluated with platelet-rich human plasma *in vitro*; the respective IC_50_ of Pt-A and Pt-B was 66 *μ*M and 203 *μ*M. Both peptides exhibited protection effects on ADP-induced paralysis in mice. After ADP administration, the paralysis time of different concentration of Pt-A and Pt-B lasted as the following: 80 mg/kg Pt-B (152.8 ± 57.8 s) < 40 mg/kg Pt-A (163.5 ± 59.8 s) < 20 mg/kg Pt-A (253.5 ± 74.5 s) < 4 mg/kg clopidogrel (a positive control, 254.5 ± 41.97 s) < 40 mg/kg Pt-B (400.8 ± 35.9 s) < 10 mg/kg Pt-A (422.8 ± 55.4 s), all of which were statistically shorter than the saline treatment (666 ± 28 s). Pulmonary tissue biopsy confirmed that Pt-A and Pt-B prevented the formation of thrombi in the lung. Unlike ADP injection alone, which caused significant reduction of peripheral platelet count, Pt-A treatment prevented the drop of peripheral platelet counts; interestingly, Pt-B could not, even though the same amount of Pt-B also showed protection effects on ADP-induced paralysis and thrombosis. More importantly, intravenous injection of Pt-A and Pt-B did not significantly increase the hemorrhage risks as clopidogrel.

## 1. Introduction

Thrombosis, associated with blood coagulation, is one of the important factors leading to cardiovascular diseases (CVD), the major cause of death worldwide [1]. Antithrombosis therapies, such as aspirin and clopidogrel in clinical practice, have contributed to dramatic improvements of patient survival, but such drugs often increase hemorrhage risks in patients [2].

In China, snake wine or snake venom liquor, prepared by infusing whole snake or its venom into grain alcohol for months, is supplied as a traditional Chinese medicine (TCM) for CVD prevention and rehabilitation [3, 4]. These kinds of liquor are used widely in Asian countries, such as Cambodia, China, Japan, Korea, Laos, Thailand, and Vietnam [5, 6]. Clinical practice suggests that the venom liquor could help patients rehabilitate from hemiplegic stroke, cervical spondylosis, scapulohumeral periarthritis, lumbar, arthritis, rheumatism, breast hyperplasia, and gout without any adverse effects [3]. Numerous modern studies have illuminated that a few specific proteins, oligopeptides, and polypeptides, in the venom present the antithrombosis and antiplatelet aggregation activities [7].

*Deinagkistrodon acutus*, a specific venomous snake in China, is commonly used to prepare medicinal liquor [3] documented in the book* Master Lei's Discourse on Drug Processing* (*Leigong Pao Jiu Lun*) initially. As described,* D. acutus* liquor could achieve the patency of blood flow and meridians. However, little is known on what the venom liquor consists of and how does it function. We hypothesize that peptides are the key components for the clinical effect of the venom liquor. In the current study, two oligopeptides were isolated from the venom liquor and sequenced. Their antiplatelet aggregation and antithrombosis efficiency were examined* in vitro* and* in vivo*. Our results suggest that Pt-A and Pt-B can prevent platelet aggregation via different mechanisms.

## 2. Materials and Methods

### 2.1. Materials

Lyophilized venom of* D. acutus* was provided by Taizhou Snake Farm (Zhejiang, China) and kept in refrigerator at −20°C. ICR mice, 18 weeks of age and 23−25 g of weight, were obtained from Laboratory Animal Center of Zhejiang Chinese Medical University (Hangzhou, China). ADP was purchased from Sigma-Aldrich (St. Louis, MO, USA); clopidogrel hydrogen sulphate tablets were from Sanofi Winthrop Industrie (Paris, France). Acetonitrile and trifluoroacetic acid (HPLC grade) were from Anaqua Chemicals Supply Inc. Limited (Houston, USA). Other chemical reagents were from China National Pharmaceutical Group Corporation (Shanghai, China). Distilled water was produced by an automatic pure water distillation (Shanghai Yarong Biochemical Instrument Company, Shanghai, China). The AKTA Explorer 10 FPLC was from GE-Amersham Pharmacia Biotech (Piscataway, USA) and Sephadex G-50 was from Pharmacia (Uppsala, Sweden). The Dionex UltiMate 3000 high performance liquid chromatography system and a Dionex Acclaim 120 C_18_ column (250 × 10 mm, 2.2 *μ*m) were purchased from Thermo Fisher Scientific Inc. (Sunnyvale, USA). AggRAM aggregometer, Sysmex XS-500i Analyzer, SpectraMax M3 Multimode Microplate Reader, and Q-TOF 2 mass spectrometer with MassLynx NT were from Helena Laboratories (Texas, USA), Sysmex Corporation (Kobe, Japan), Molecular Devices, Inc. (Sunnyvale, USA), and Micromass UK Ltd. (Altrincham, UK), respectively. The Pt-A and Pt-B peptides were synthesized by Shanghai Ketai Bio-Science and Technology Co. Ltd. (Shanghai, China).

### 2.2. Preparation of Snake Venom Liquor

500 mg venom was infused in 1 L 60% ethanol for 3 months at room temperature. The liquor was centrifuged at 12,000 g for 30 min at 4°C. The debris was washed with 300 mL 60% ethanol and discarded; the supernatants were pooled and lyophilized at 60°C.

### 2.3. Isolation and Identification of the Two Peptides

The lyophilized material was resuspended with distilled demineralized water (ddH_2_O) and applied to a water-equilibrated Sephadex G-50 column (38 cm × 1.6 cm) of the AKTA Explorer FPLC and monitored at 215 nm. The target peptides were eluted together with ddH_2_O at room temperature with the flow rate of 2 mL/min. This eluent was applied to semipreparative RP-HPLC, monitored at 215 nm, on an Acclaim 120 C_18_ column (250 × 10 mm) equilibrated with buffer A (5% acetonitrile containing 0.1% trifluoroacetic acid). The two peptides were eluted and separated with an acetonitrile gradient with its concentrations changed from 5% in buffer A to 35% in buffer B (35% acetonitrile containing 0.1% trifluoroacetic acid) in 30 min. The column temperature was kept at 30°C. The amino acid sequence of peptides was identified with Q-TOF mass spectrometer and NMR in comparison with standards [8, 9].

### 2.4. Antiplatelet Aggregation Activity* In Vitro*

The antiplatelet activity of Pt-A and Pt-B was evaluated in ADP-induced platelet aggregation assays described by Born [10]. The venous blood was from healthy volunteers free from aspirin or other nonsteroidal anti-inflammatory drugs for more than 7 days and kept in citrated tubes. The platelets were precipitated by centrifugation at 200 ×g for 8 min and resuspended in platelet poor serum, resulting in a final concentration of 2.5–3.0 × 10^8^ cells/mL. 250 *µ*L venous blood was incubated with 10 *µ*L 0.008, 0.086, 0.175, 0.258, 0.347, 0.508, and 0.75 mM Pt-A saline solution or 0.028, 0.083, 0.166, 0.250, 0.416, 0.583, and 0.72 mM Pt-B saline solution, respectively, at 37°C for 5 minutes. 5 *µ*L 5 *µ*M ADP was pipetted into the reaction and the maximal aggregation of platelets was monitored with Helena Platelet Aggregation Analyzer in 5 minutes. The assays were performed in triplicate and the efficiency was present as median lethal dose (IC_50_).

### 2.5. Protection Efficiency of Pt-A and Pt-B on ADP-Induced Pulmonary Thromboembolism in Mice

64 male ICR mice (18 months old, 23–25 g) were randomly divided into 8 groups. These mice were pretreated with intravenous injection of Pt-A (10, 20, or 40 mg/kg), Pt-B (20, 40, or 80 mg/kg), clopidogrel (4 mg/kg, as a positive control), or saline solution (as a negative control) in tail vein. Fifteen minutes later, the mice were paralyzed by intravenous injection of ADP (200 mg/kg). The time when the mice come back to movement was recorded as the paralysis time, and it was compared and analyzed statistically [11, 12]. Another 63 male ICR mice (18 months old, 23–25 g) were divided into 7 groups (without clopidogrel group) and treated as described above again. The peripheral blood was collected 1 minute after ADP administration for platelet counting with Sysmex XS-500i Hematology Analyzer. Meanwhile, the lung was fixed in 10% formalin. The paraffin-embedded sections of lungs were stained with hematoxylin-eosin staining and observed under microscope [13].

### 2.6. Haemorrhage Test

The haemorrhage efficiency of the two peptides was identified with a modified tail cutting method [14]. Male ICR mice were randomly grouped with 7 mice in each group and preprotected with Pt-A (40 mg/kg), Pt-B (40 mg/kg), clopidogrel (4 mg/kg), or saline (as a control) by tail vein injection, 15 minutes (clopidogrel was 2 h) before tail cutting. The tails of mice that were anesthetized with pentobarbital sodium (100 mg/kg) were transected 5 mm from the tip and stretched into 12 mL saline for 10 minutes at 37°C. The same volume red blood cell lysis buffer (15 mM NH_4_Cl, 10 mM KHCO_3_, and 0.1 mM Na_2_EDTA [disodium ethylenediamine tetraacetate], pH = 7.3) was added and mixed well. Meanwhile, a group of standard mixtures were prepared. The absorbance of these solutions was estimated at 490 nm and the blood volumes were calculated with standard curve [15, 16].

### 2.7. Statistical Analysis

Results were presented as means ± SD obtained from the indicated number of animals or samples. The statistical analysis was performed by GraphPad Prism 5.00, using unpaired Student's *t*-test.

## 3. Results

### 3.1. Purification and Identification of Pt-A and Pt-B

**Figure 1:**
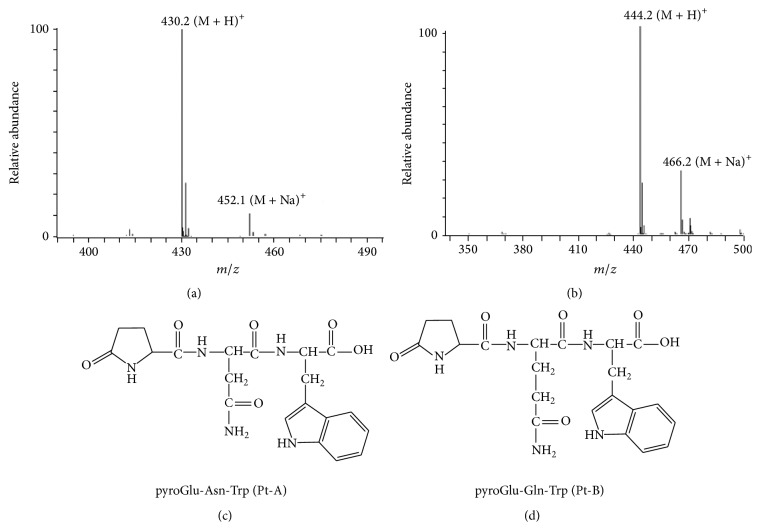
Q-TOF mass spectra of HPLC separated Pt-A (a) and Pt-B (b). The molecular masses of Pt-A and Pt-B were 429 Da (a) and 443 Da (b), as identified with Q-TOF mass analysis. The amino acid sequence of Pt-A (c) and Pt-B (d) was identified through comparing their Q-TOF spectra to those of synthesized tripeptides (positive controls were not shown).

Pt-A and Pt-B were purified with two chromatographical steps; their yields from 0.5 g venom were 1 mg/g and 11 mg/g, separately. The water-soluble fraction in snake venom liquor was 0.232 g and in the Sephadex G-50 eluent was 17.95 mg. The retention times of purified Pt-A and Pt-B were identified to those of synthesized oligopeptides pyroglutamate-asparagine-tryptophan (PAT) and pyroglutamate-glutamine-tryptophan (PGT), respectively (results not shown). And, based on ion peaks at *m*/*z* 430.2 ([M + H]^+^) and 444.2 ([M + H]^+^), the molecular masses of Pt-A and Pt-B (Figure 1) were determined to be 429 Da and 443 Da, which were identified to those of synthesized PAT and PGT, also.

### 3.2. Antiplatelet Aggregation Efficiency* In Vitro*

**Figure 2:**
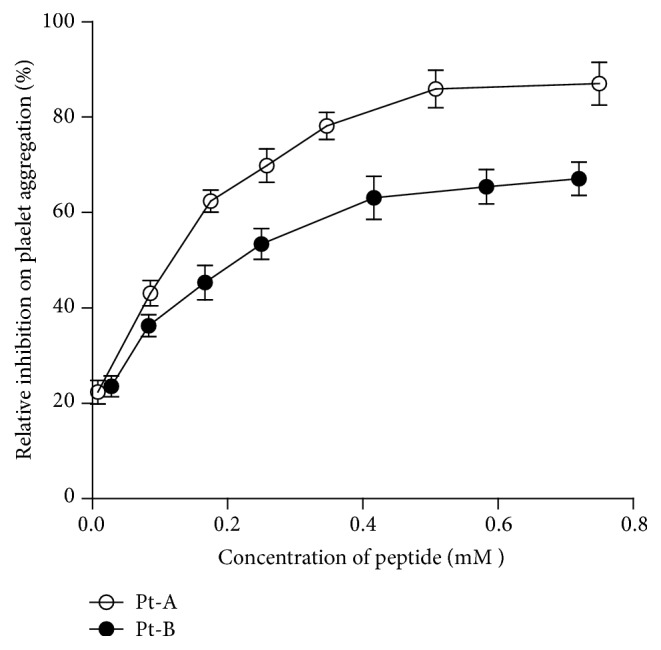
Relative inhibitory efficiency of Pt-A and Pt-B on ADP-induced platelet aggregation. The platelets were preincubated with Pt-A, Pt-B, or saline at 37°C for 5 min first. Platelet aggregation was initiated with 5 *µ*M ADP and monitored with Platelet Aggregation Analyzer. The relative inhibitory efficiencies of various concentrations of Pt-A (empty circles) and Pt-B (solid circles) were normalized with the saline group and presented as the mean ± SD (*n* = 3).

The anti-human platelet aggregation activity of the two tripeptides, in a dose-dependent manner, was shown in Figure 2. Both Pt-A and Pt-B inhibited platelet aggregation in the* in vitro* experiment, but Pt-A had a stronger effect than Pt-B. IC_50_ of Pt-A and Pt-B was 0.066 mM and 0.203 mM, respectively.

### 3.3. Protection Efficiency of Pt-A and Pt-B on ADP-Induced Thrombosis Mice Model

**Figure 3:**
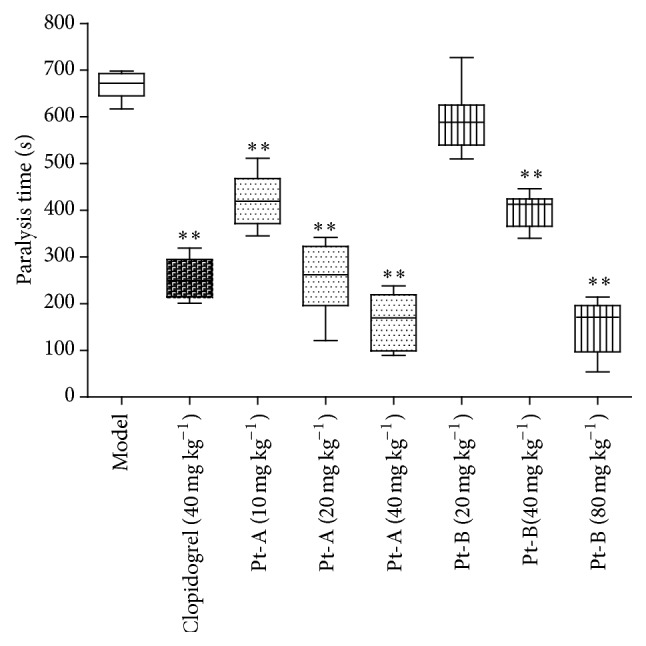
Effects of Pt-A and Pt-B on paralysis that was induced by ADP in mice. The mice in each group were injected intravenously (IV) with the following: saline; clopidogrel (40 mg kg^−1^), a positive control; Pt-A (10 mg kg^−1^, 20 mg kg^−1^, and 40 mg kg^−1^) and Pt-B (20 mg kg^−1^, 40 mg kg^−1^, and 80 mg kg^−1^). After 15 min, ADP (200 mg kg^−1^) was IV injected to induce paralysis. The time that the mice come back to move was recorded as the paralysis time, which was presented as the mean ± SD. All the data were compared to the saline group and analyzed by unpaired Student's *t*-test. ^*∗∗*^ *P* < 0.01.

The ADP-induced thrombosis in mice was one of the most used animal models for antithrombotic agent evaluation with indicators including the paralysis in the animals, reduction of free platelet cells in the blood, and the presence of micro-thrombi in the lung. In the current study, ADP paralyzed the mice in the saline group for 666 ± 28 seconds (mean ± SD). In comparison, the paralysis time of Pt-A at the doses of 10, 20, and 40 mg/kg and Pt-B at the doses of 40 and 80 mg/kg groups lasted for 422.8 ± 55.4, 253.5 ± 74.5, 163.5 ± 59.8, 400.8 ± 35.9, and 152.8 ± 57.8 seconds, respectively, all of which were statistically significantly shorter compared with the time in the saline group (*P* < 0.01). Simultaneously, the mice of clopidogrel (4 mg/kg) group, a positive control, were paralyzed for 254.5 ± 41.97 seconds. However, the paralysis time of 20 mg/kg Pt-B group was 592.8 ± 67.9 seconds, showing no difference from the saline group (*P* > 0.05). This suggested that pretreatment with Pt-A (10, 20, and 40 mg/kg) and Pt-B (40, 80 mg/kg) protected mice from ADP-induced paralysis significantly (Figure 3).

When the mice in the control group were pretreated with saline and injected with saline 15 minutes later, the mean count of their platelets was 1.00 × 10^6^/*μ*L. Injection of ADP after saline pretreatment (the paralysis group) significantly decreased the peripheral platelet count to 0.45 × 10^6^/*μ*L. In contrast, pretreatment with 10, 20, and 40 mg/kg Pt-A kept the platelet counts to 0.87 × 10^6^, 1.03 × 10^6^, and 0.96 × 10^6^/*μ*L, similar levels to the control group. Interestingly, even though 40 and 80 mg/kg Pt-B pretreatment presented similar protection effects on the ADP-induced paralysis as 10 mg/kg and 40 mg/kg Pt-A, such amounts of Pt-B could not prevent the ADP-induced reduction of platelet counts (0.514 × 10^6^ and 0.69 × 10^6^/*μ*L, resp.) (Table 1). Our results demonstrated that only Pt-A presented significant protection effects to the ADP-induced reduction of platelets (*P* > 0.05).

**Figure 4:**
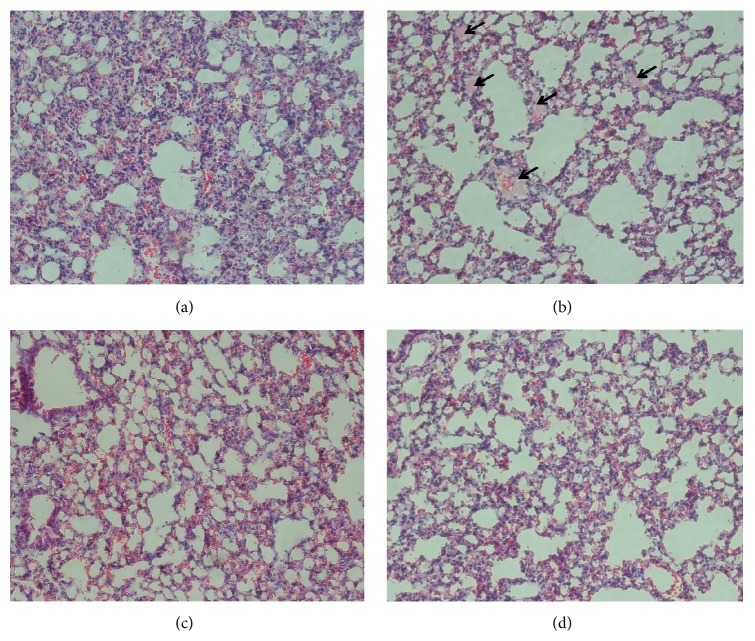
Protection effect of Pt-A and Pt-B on the ADP-induced formation of acute pulmonary thromboembolism in mice. The micro-thrombi (arrow) in the lung of (a) clopidogrel, (c) Pt-A 40 mg kg^−1^, (d) Pt-B 40 mg kg^−1^, or (b) saline pretreated mice were photographed (magnification ×20).

Next, we observed the formation of micro-thrombi under microscope in the lung from the same experiment (Figure 4). The homogeneous structure micro-thrombi, stained with hematoxylin-eosin staining in pink (marked with arrows), could be only observed in the paralysis control group (Figure 4(b)) but not in the negative control (Figure 4(a)), 40 mg/kg Pt-A (Figure 4(c)), and 40 mg/kg Pt-B (Figure 4(d)) groups.

### 3.4. Potential Haemorrhage Risk

**Figure 5:**
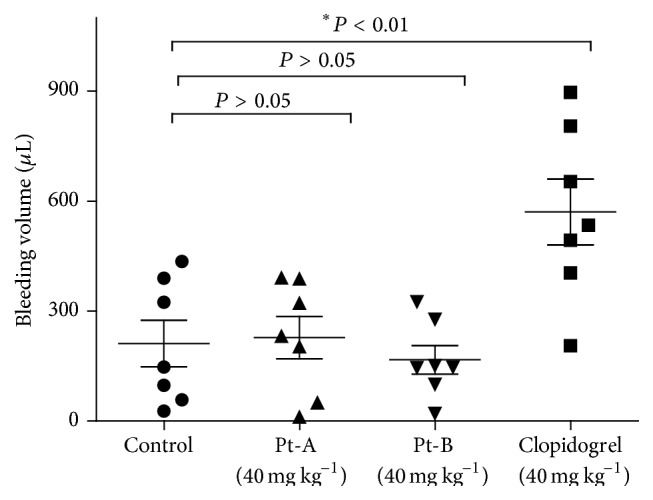
Effects of Pt-A and Pt-B on haemorrhage risks in comparison with clopidogrel. Mice in each group received the corresponding chemicals, and the control group received saline. Each dot represented the bleeding volume of an individual mouse. Means ± SD of each group were also shown. The data was analyzed with unpaired Student's *t*-test and *P* < 0.05 was considered as statistically significant. The significant one was marked with “*∗*”

An increased bleeding risk is mostly associated with antiplatelet aggregation and antithrombosis agents, for example, clopidogrel. To assess the bleeding risk, the loss of blood in Pt-A, Pt-B, clopidogrel, and saline (control) pretreated groups was in comparison measured (Figure 5). As expected, mice in the clopidogrel group lost more blood than those in the control (570.1 ± 237 *μ*L versus 211.1 ± 168 *μ*L; *P* < 0.01). However, no significant difference of bleeding volume was observed between the two peptides groups (40 mg/kg) and the control (Pt-A 227.6 ± 156.8 *μ*L versus 211.1 ± 168 *μ*L, Pt-B 166.9 ± 103.5 *μ*L versus 211.1 ± 168 *μ*L; (*P* > 0.05)). This suggested that applying of Pt-A and Pt-B (40 mg/kg) to protect ADP-induced pulmonary thrombosis reduced the risk of bleeding in mice.

## 4. Discussion

Snake, the symbol of guilds of medicine and pharmacy in Europe, implied restoration of the health from sickness and bringing the dead back to life. Across China, as well as other Asian countries, snake and snake venom are recognized as a traditional medicine. The main components of venom are proteins and peptides. The clinical significance of its different components is diverse and sometimes even they contradict each other, but as a whole they work fast and synergistically [17]. The multiple facets and functions of snake venom make its components so valuable for medicine.

*Deinagkistrodon acutus*, a specific genus of Viperidae family in Southeast Asia, produces and secretes lethal venom. The venom liquor exhibits significant clinical effect on CVD [3]. In present study, we isolated Pt-A (pGlu-Asn-Trp) and Pt-B (pGlu-Gln-Trp) from the* D. acutus* venom liquor and demonstrated the antiplatelet aggregation and antithrombosis effects of them. In previous studies, Pt-A was found in* Bungarus* (1973) [18],* Bothrops asper* (1993) [19], and two* Trimeresurus* species (1993, 1998) [20, 21]. Henceforth, another two highly homogenetic peptides, Glu-Gln-Trp (Pt-B) and Glu-Lys-Trp (pEKW), have been isolated simultaneously. In 2009, Pt-A was isolated from* D. acutus* venom for the first time by Kong et al. [9]. But no trace of Pt-B was observed in this species. To the best of our knowledge, it is the first study to illuminate Pt-B in* D. acutus* venom. However no trace of pEKW was observed in our research.

Even though Pt-A and Pt-B were discovered for years (1966), little of their potential clinical characters were studied. Majority of studies focused on the multiple metalloproteinases inhibitory effect of Pt-A and Pt-B [19, 21]. The platelet aggregation inhibitory activity of Pt-A on rabbit* in vitro* was demonstrated initially by Xiong et al. [14], with IC_50_ of 178 *μ*M. To our surprise, IC_50_ of Pt-A on anti-human platelet aggregation was 66 *μ*M* in vitro*, much more efficient than that of Pt-B (IC_50_ = 203 *μ*M). In addition, Pt-A exerted significant anti-ADP-induced platelet aggregation activity* in vivo* and Pt-B did not (Table 1). Surprisingly, Pt-B as well as Pt-A and clopidogrel could protect mice from ADP-stimulated paralysis (Figure 2) and the generation of microvascular thrombosis in the lung (Figure 3). It is not clear how Pt-B achieved its protection function against ADP-induced paralysis without exhibiting antiplatelet aggregation activity* in vivo*. We speculate that Pt-B takes its antithrombosis function via a different mechanism from Pt-A. The antiplatelet aggregation and antithrombosis function of Pt-A were consistent with the research conclusion of Pt-A in the arteriovenous shunt models and inferior vena ligation models [9].

Besides, the hemorrhagic risks of Pt-A and Pt-B were compared with clopidogrel in a modified haemorrhage assay. The bleeding volumes in the two tripeptides pretreated groups were comparable to the saline group. On the contrary, the mice in the clopidogrel group lost more blood (Figure 5). This result demonstrates that Pt-A and Pt-B can prohibit platelet aggregation and thrombus formation without increasing the hemorrhagic risks.

To conclude, this study suggests that Pt-A and Pt-B are the bioactive components in the snake venom liquor of* Deinagkistrodon acutus* that contributed to cardiovascular disease prevention. These two tripeptides could be potential candidates to be implemented as nutraceuticals and pharmaceuticals against thrombosis and its related diseases. In addition, it is expected that this study will develop interests in research and potential applications of snake venom liquor.

## Figures and Tables

**Table 1:** The platelet counts in mice blood (×10^6^/mL).

	Control group	ADP-induced group	Pt-A (10 mg/kg)	Pt-A (20 mg/kg)	Pt-A (40 mg/kg)	Pt-B (20 mg/kg)	Pt-B (40 mg/kg)	Pt-B (80 mg/kg)
Mean ± SD	1003 ± 123.3	454.3 ± 90.21	868.7 ± 146.8	1032 ± 123.4	959 ± 89.9	489.6 ± 121.8	514.6 ± 97.16	690.1 ± 132.6
*P* (versus control group)		^*∗*^ *P* < 0.01	*P* > 0.05	*P* > 0.05	*P* > 0.05	^*∗*^ *P* < 0.01	^*∗*^ *P* < 0.01	^*∗*^ *P* < 0.01

The peripheral platelets were counted with Sysmex XS-500i Hematology Analyzer after ADP injection. Values were presented as mean ± SD with *n* = 8 of each group. The data were analyzed by unpaired Student's *t*-test. If *P* < 0.05, it was regarded as significant. The significant one was marked with “*∗*”.
